# Fracture and Fatigue in Metals and Alloys

**DOI:** 10.3390/ma19071405

**Published:** 2026-04-01

**Authors:** Shengci Li, Zhigang Wang

**Affiliations:** 1 School of Materials Science and Engineering, Jiangxi University of Science and Technology, Ganzhou 341000, China; 2 State Key Laboratory of Nonferrous Structural Materials, Jiangxi University of Science and Technology, Ganzhou 341000, China; 3 Jiangxi Provincial Key Laboratory of High-Performance Steel and Iron Alloy Materials, Ganzhou 341000, China

Fatigue and fracture are the main failure forms in material service [1,2,3,4,5]. Research on fatigue and fracture involves important industries and key fields such as material research and development [6], mechanical manufacturing [7], modern transportation [8], infrastructure construction [9], petrochemicals [10], and aerospace [11]. Therefore, the purpose of this Special Issue is to share the latest technological achievements and jointly explore the most pressing and complex issues in the field of fatigue and fracture. Manuscripts on many subjects regarding fracture and fatigue in metals and alloys were submitted for the current Special Issue (SI). Following the peer-review process, seven papers have been accepted for publication. These papers cover the topics of DP780 dual-phase steel, copper-based steel-backing self-lubricating materials, eccentric camshaft forging, additively manufactured Al-Mg-Si alloys, low-carbon steel, pearlite steel and Ti-4Zr-6Al-0.6Si-0.5Mo alloys fabricated via powder metallurgy.

Contribution 1 [12] revealed the microstructural evolution and stress–strain distribution of DP780 ferrite/martensite dual-phase steel during a uniaxial tensile deformation process using in situ EBSD and the crystal plastic finite element method (CPFEM). Results showed that the geometrically necessary dislocations (GND) in ferrite accumulated continuously, which is conducive to the formation of grain boundaries, and the plastic deformation mainly occurred in the soft ferrite region; interfacial debonding was caused by the accumulation of GND.

Contribution 2 [13] prepared a copper-based steel-backing material through a combination of mechanical alloying and secondary sintering methods, investigating the effect of Y_2_O_3_ content on the microstructure, hardness, and tribological properties of the copper-based self-lubricating layer. The results demonstrate that the incorporation of Y_2_O_3_ effectively improves the tribological properties of the composite material, significantly reducing wear during the friction process and decreasing the wear rate by 77%. Under the experimental conditions, the optimal Y_2_O_3_ content was determined to be 1 wt%.

Contribution 3 [14] implemented a vertical upsetting extrusion forming methodology for camshaft forging production to develop an improved process method of eliminating defect formation in forged components that involved adopting a vertical upsetting extrusion forming method with a 40° diversion angle at the junction between the first step and the thin rod in the die cavity. Numerical simulations confirmed complete elimination of deformation dead zones in the optimized process.

Contribution 4 [15] separately introduced TiB_2_ and TiC particles into an Al-Mg-Si alloy fabricated by wire-arc additive manufacturing (WAAM) to solve the problem of hot cracking. Results showed that the hot cracks were completely suppressed due to the transformation from columnar grains to fine equiaxed grains with a mean diameter of approximately 10 μm. The fatigue resistance of the heat-treated Al-Mg-Si/TiC was better than that of the heat-treated Al-Mg-Si/TiB_2_ due to lower porosity and a more uniform distribution of TiC particles.

Contribution 5 [16] investigated the kinetics and evolution of hydrogen-induced cracking (HIC), and the Monte Carlo method was used to model the interconnection of individual HIC cracks. The results indicate that HIC propagation occurs via cleavage and quasi-cleavage mechanisms, with crack interconnection by ductile shear tearing, where the driving force for HIC is the accumulated hydrogen pressure within the internal HIC cracks, explaining why the crack growth rates are nearly constant in each stage of HIC growth.

Contribution 6 [17] studied the serviceability of pearlite (R260) steel welded joints (WJs) during long-term operation in hydrogen-containing environments for application in additive manufacturing technology. Results show that in hydrogen-saturated (up to 4.7 ppm) specimens, the desired fatigue crack can be obtained at a considerably lower number of cycles of the same dynamic load than in non-hydrogenated ones; critical fracture occurs faster in hydrogenated specimens (46.6 MPa m^0.5^) than in non-hydrogenated ones.

Contribution 7 [18] investigated the hot deformation behavior of a Ti-4Zr-6Al-0.6Si-0.5Mo high-temperature titanium alloy under temperatures of 800–1100 °C, strain rates of 0.001–1 s^−1^, and a true strain of 0.5. The results show that the optimal hot processing window for the alloy is a deformation temperature of 950–1100 °C and a strain rate of 0.001–0.01 s^−1^. Flow instability occurred within two domains—800–850 °C at strain rates of 0.1–1 s^−1^ and 900–1075 °C at strain rates of 0.01–0.1 s^−1^—where the alloy is prone to cracking, resulting in processing failure.

In summary, the studies in this Special Issue provide a comprehensive overview of the current innovations in the field of fracture and fatigue in metals and alloys. The current Special Issue is a collection of research contributions covering microstructure evolution and strain localization, tribological properties, defect analysis during forging, hot cracks in wire-arc additive manufacturing, hydrogen-induced cracking, hydrogen degradation, and powder metallurgy. Both experimental and numerical simulations were reported in different papers.
